# Editorial: Future-proofing the workforce for person-centered healthcare

**DOI:** 10.3389/frhs.2026.1963260

**Published:** 2026-08-27

**Authors:** Paul Slater, Rebekkah Middleton

**Affiliations:** 1 Faculty of Life & Health Sciences, Ulster University, Coleraine, United Kingdom; 2 Faculty of Science Medicine and Health, University of Wollongong, Wollongong, NSW, Australia

**Keywords:** editorial, future-proofing, healthcare, person-centered, workforce

Healthcare systems worldwide face what may be their greatest challenge of the twenty-first century: sustaining a workforce capable of delivering high-quality, compassionate care (1). Workforce shortages, rising turnover, burnout and declining staff engagement have become persistent features of health systems across countries and care settings (2). While recruitment remains important (3), growing evidence suggests that the future of healthcare depends not only on attracting new health professionals but also on creating workplace environments where individuals choose to remain, develop and flourish throughout their careers (4, 5).

The papers in this Research Topic collectively highlight a critical shift in thinking about workforce sustainability. Rather than framing workforce shortages solely as a staffing problem, they position them as a cultural challenge (3, Cardiff and Frost, Croston et al., Golz et al.). Across diverse contexts, including acute care, residential aged care, HIV services and healthcare education, a consistent finding emerges: organisational cultures that support meaningful relationships, professional autonomy, shared purpose and person-centred ways of working are associated with improved staff wellbeing, stronger engagement and greater workforce stability.

Person-centredness is central to future-proofing the healthcare workforce. International policy increasingly identifies person-centred practice as both a quality standard and a workforce strategy (5–7). Beyond its well-established benefits for patient experience and healthful outcomes, person-centredness creates conditions that enable healthcare professionals to work in ways that align with their values and professional identities (6, 8). By fostering collaboration, shared decision-making, reflective practice and authentic relationships, person-centred cultures support resilience and adaptability in increasingly complex healthcare environments (6).

Furthermore, person-centredness aligns closely with contemporary workforce policy emphasising value-based healthcare (9, 10). Rather than measuring success solely through clinical activity or service volume, value-based approaches prioritise outcomes that matter to patients alongside efficient use of healthcare resources. Person-centred practice therefore becomes both a quality improvement and workforce strategy by enabling healthcare professionals to deliver care that is more responsive, efficient, and sustainable.

A central message emerging from this Research Topic of papers is that retention should not be viewed as a stand-alone human resources objective. Instead, retention appears to be a consequence of (supportive or unsupportive) organisational culture. Healthcare professionals often leave organisations not simply because of workload or financial considerations but because of cumulative experiences of disconnection, lack of recognition and an inability to practise according to their professional values. Efforts to improve retention therefore require attention to the broader conditions that shape everyday work experiences (Bills and Gale).

Leadership is particularly influential in this regard. The studies represented in this Research Topic (Cardiff and Frost, Golz et al.) reinforce contemporary understandings of leadership as a relational and cultural process rather than a hierarchical function. Leadership practices that promote psychological safety, trust, shared purpose and professional growth are consistently associated with more positive workforce experiences. Importantly, leadership development is not presented merely as the acquisition of managerial competencies but as a means of shaping cultures that enable individuals and teams to thrive (Cardiff and Frost). At the same time, the evidence reminds us that leadership alone cannot compensate for excessive workloads, resource constraints or poorly designed healthcare systems. Sustainable workforce strategies require both cultural and structural investment (Golz et al.).

The importance of a person-centred workplace culture extends beyond staff wellbeing to the quality of care that patients and families receive (6). Organisations characterised by authentic relationships, supportive practice environments and shared values appear better able to sustain both workforce engagement and person-centred care (Marriott-Stratham et al., Wang et al.). Conversely, rigid systems, transactional approaches to management and depersonalising organisational structures can undermine both workforce wellbeing and care quality (Wallner et al.). These findings reinforce the growing recognition that workforce experience and patient experience are closely interconnected rather than separate organisational priorities.

Education also emerges as a critical component of workforce sustainability (Wang et al., Baldie et al.). Preparing the future healthcare workforce requires more than the development of technical expertise. Key skills such as communication, emotional intelligence, cultural responsiveness, teamwork, compassion and reflective practice are increasingly recognised as essential capabilities for contemporary healthcare professionals (Baldie et al.). As healthcare systems become more integrated, technologically enabled and community focused, these relational capabilities will be as important as clinical competence in determining the quality and sustainability of care.

An important challenge highlighted across the Research Topic concerns measurement and what is important to measure. Healthcare systems have become highly effective at measuring activity, productivity and performance, yet remain less successful at evaluating the relationships, cultures and caring behaviours that underpin person-centred care. What organisations choose to measure inevitably influences what they prioritise. Performance systems that focus primarily on task completion risk overlooking the relational dimensions of practice that contribute to staff wellbeing, patient experience and long-term workforce stability. Developing meaningful measures of culture, compassion, engagement and person-centredness should therefore be considered a strategic workforce priority.

Collectively, the papers encourage a move beyond the language of retention towards the broader concept of workforce flourishing. Flourishing extends beyond simply preventing staff from leaving. It encompasses meaningful work, supportive leadership, psychological safety, professional growth, strong workplace relationships and alignment between individual(s) and organisational values. Increasingly, these factors are being recognised not as aspirational ideals but as measurable determinants of workforce stability, organisational performance and care quality.

The workforce challenges confronting healthcare systems are complex, and no single intervention will provide a panacea. Nevertheless, the evidence presented in this Research Topic offers a coherent and optimistic message. From the research across healthcare settings, health professionals and methodological approaches, similar conclusions emerge: Organisations that invest in people, relationships and culture appear better positioned to retain experienced health professionals, support staff wellbeing and deliver high-quality care. For those involved in research in this area, this message is not new but needs to be acted upon. The challenge is in implementing it (Croston et al.).

Ultimately, the future of healthcare will depend not only on how many professionals enter the workforce but on whether healthcare organisations create cultures in which people can thrive, contribute meaningfully and build long, fulfilling and sustainable careers. In that regard, enabling workforce flourishing may be one of the most important healthcare reforms of our time.
